# A nano-mechanical instability as primary contribution to rolling resistance

**DOI:** 10.1038/s41598-017-11728-6

**Published:** 2017-09-12

**Authors:** Jan Meyer, Reinhard Hentschke, Jonathan Hager, Nils W. Hojdis, Hossein Ali Karimi-Varzaneh

**Affiliations:** 1 School of Mathematics and Natural Sciences, Bergische Universität, D-42097 Wuppertal, Germany; 2 Continental Reifen Deutschland GmbH, D-30419 Hannover, Germany

**Keywords:** Mechanical engineering, Polymers, Atomistic models

## Abstract

Rolling resistance ranks among the top ten automobile megatrends, because it is directly linked to fuel efficiency and emissions reduction. The mechanisms controlling this phenomenon are hidden deeply inside the complexity of tire tread materials and do elude direct experimental observation. Here we use atomistic molecular modelling to identify a novel nano-mechanical mechanism for dissipative loss in silica filled elastomers when the latter are subjected to dynamic strain. The force-vs-particle separation curve of a single silica particle-to-silica particle contact, embedded inside a polyisoprene rubber matrix, is obtained, while the contact is opened and closed by a cyclic force. We confirm the occurrence of spontaneous relative displacements (‘jolts’) of the filler particles. These jolts give rise to energy dissipation in addition to the usual viscous loss in the polymer matrix. As the temperature is increased the new loss mechanism becomes dominant. This has important technical implications for the control and reduction of tire rolling resistance as well as for many other elastomer composite applications involving dynamic loading.

## Introduction

**Figure 1:**
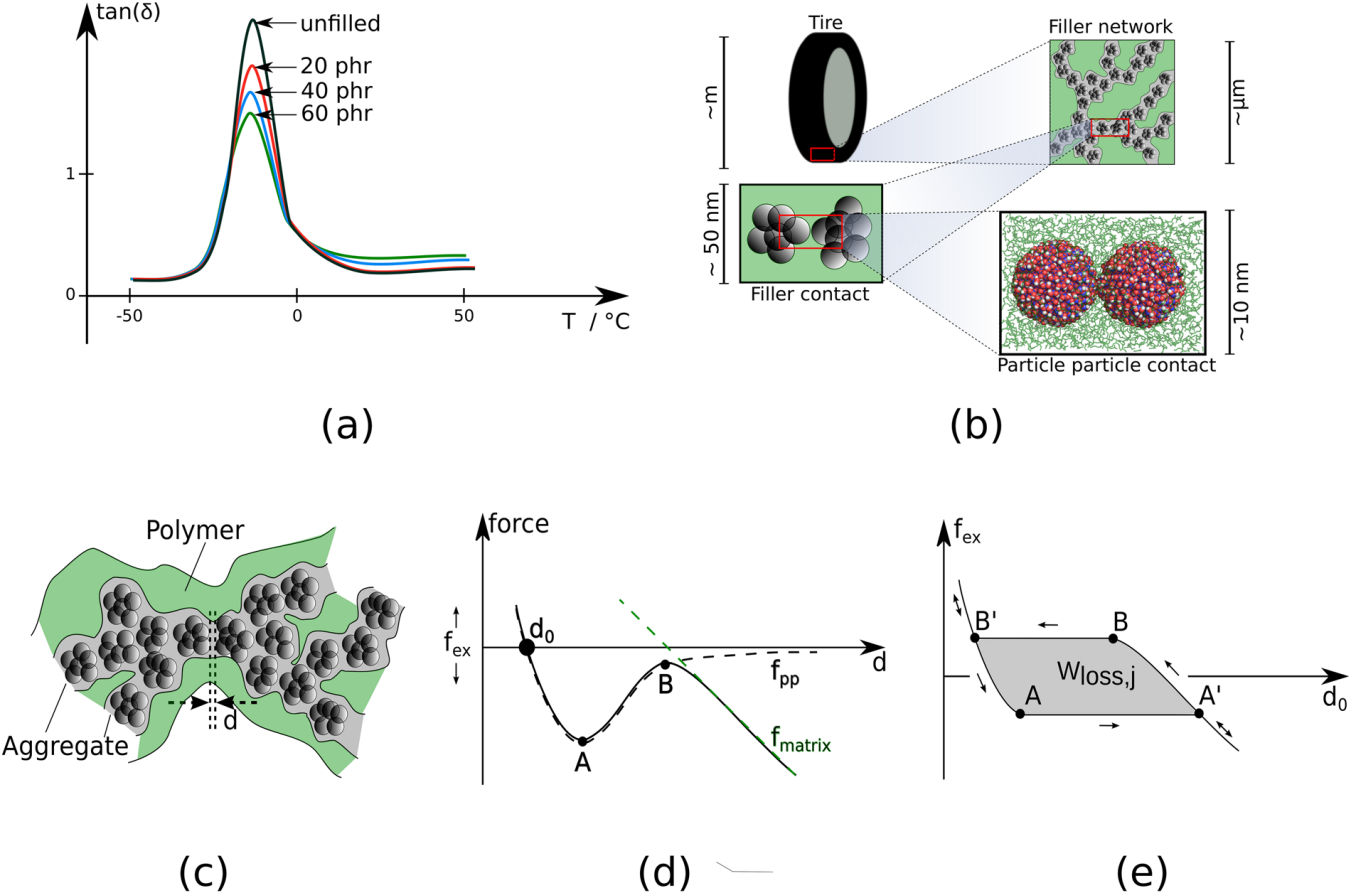
(**a**) Sketch of tan *δ* vs. temperature comparing the unfilled to the filled elastomer (filler content in phr: parts [by weight] per hundred rubber). (**b**) The figure illustrates the hierarchy of scales inside a filled elastomer, here represented by the sketch of a tire. The highest resolution depicts a partial molecular dynamics simulation snapshot showing two silica model particles embedded in 85 polyisoprene chains of length 200 (monomers). (**c**) Cartoon of a filler network branch consisting of aggregates containing (primary) filler particles. For one of the aggregate-to-aggregate contacts the particle-to-particle separation, *d*, is indicated. (**d**) The two contributions (dashed lines) to the total inter-aggregate force curve (solid line). (**e**) The path in the *f*_*pp*_(*d*) + *f*_*matrix*_(*d*)-d-plane when *d* = *d*_*o*_. Note that the shaded area is the dissipated work.

Technical rubbers are nano-composites containing large amounts of filler. The filler nanoparticles form branched percolating networks, embedded in the polymer matrix, enabling the material to support large dynamic loads over millions of load cycles. The mechanics of these composite materials is complex (e.g. refs 1–3). Here it is their highly non-linear behaviour on which we focus. An effect of major importance in this context was extensively studied in the nineteen sixties by A. R. Payne, even though its original observation dates back to the early 1940s^4^. The Payne effect (e.g. refs 5–8) describes the pronounced decrease of the storage modulus, *μ*′(*u*), with increasing strain amplitude, *u*, in filled rubbers during cyclic loading. Because the effect does not occur in unfilled rubbers, its cause must be related to either the rubber-filler or the filler-filler interface(s). The rapid decrease of *μ*′(*u*) with increasing *u* is accompanied by a pronounced maximum of the loss modulus, *μ*′′(*u*). If both *μ*′(*u*) and *μ*′′(*u*) are measured at fixed *u*, but variable temperature, *T*, i.e. *μ*′ = *μ*′(*u*, *T*) and *μ*′′ = *μ*′′(*u*, *T*), the presence of the filler network leads to an increase on the high temperature side of the tan *δ*-peak, where tan *δ* ≡ *μ*′′/*μ*′. This is depicted in Fig. 1(a). Examples of actual corresponding data are shown in Fig. 8 of ref. 9, Fig. 6 of ref. 10. or Figs 2.63 and 2.64 of ref. 3. Qualitatively the effect is independent of the type of the nano-filler. This is different in the limit of low polymer crosslink density, which permits the polymer to ‘flow’ (e.g. ref. 11). The tan *δ*-peak as such is associated with the glass transition of the polymer. But other sections along the tan *δ*-vs-T curve serve as important laboratory indicators for various technical performance parameters. Empirical studies have shown that tan *δ* at temperatures close to or exceeding 50 °C is a very good indicator for rolling resistance and thus for the energy efficiency of an automobile tire^12–14^. It is important to note that rolling resistance is not governed by the immediate contact between tire tread and the road surface but occurs deep inside the rubber compound when the latter is deformed^13^. Understanding the molecular mechanism of dissipative loss in the aforementioned temperature regime therefore is a key step towards the control of rolling resistance. We stress however that the following discussion is not limited to rolling resistance. This is because the same mechanism is present in the numerous other applications of elastomer composite materials involving dynamic loading.

The present molecular modelling study of a single filler particle contact embedded in a polyisoprene matrix is a ‘proof of principle’ study. It suggests that a novel dissipative mechanism primarily is responsible for rolling resistance in highly filled rubber. The material of interest here is an elastomer which contains active filler, forming a network throughout the elastomer. A pictorial illustration is shown in Fig. 1(b). Note that a certain fraction of the network’s filler strands will be load-bearing if the sample is subjected to dynamic deformation. The basic structural elements along these strands are contacts formed by adjacent aggregates of primary filler particles embedded in polymer. In the present simulation study, the two spherical silica particles represent filler aggregates (cf. the lower right panel in Fig. 1(b)). While keeping the position of the left particle fixed, the right particle is displaced by a given force. Subsequently the resulting force-vs-displacement curves are analysed and the attendant dissipation is determined. This computer experiment is carried out at different temperatures. We find that at sufficiently high temperatures the dissipation is predominantly due to a jolt-like motion of the particles relative to each other. These jolts occur because of the special functional form of the total interaction. We conclude that this nano-mechanical instability is the major contribution to rolling resistance in automobile tire treads.

## Results

### The jump-in-jump-out mechanism

A likely candidate mechanism for dissipative loss due to filler-filler interaction is the jump-in-jump-out mechanism originally suggested in ref. 15. A pictorial illustration of this mechanism is shown in the panels (c) through (e) of Fig. 1. The basic element is a contact between filler aggregates within a load-bearing filler network strand embedded inside a polymer matrix (Fig. 1(c)). The closest separation between the adjacent aggregates is *d*. The force between the two aggregates as function of *d* comprises two basic contributions (Fig. 1(d)). The first one, *f*_*pp*_(*d*), is the direct interaction between the aggregates, which is repulsive when the aggregates begin to overlap and attractive when *d* is sufficiently large. Attractive interactions are contributed by non-covalent bonds between aggregate surfaces. Examples include polymer segments extending from one surface to the other or hydrogen bonds if the filler is silica. Generally, all conceivable non-covalent molecular interactions fall into this category. In addition, there are attractive long-range dispersion forces between the aggregates as a whole (cf. ref. 16), which are of lesser importance here. The second force contribution, *f*_*matrix*_(*d*), is due to the conformational entropy reduction when polymer segments between cross-links are stretched (rubber elasticity). Note that the sum of the two contributions is the S-shaped solid line in Fig. 1(d). The equilibrium value of *d*, here denoted as *d*_*o*_, is the root of 0 = *f*_*pp*_(*d*) + *f*_*matrix*_(*d*). In ref. 15 the authors assume that an external force applied to a filled elastomer sample gives rise to the average additional force *f*_*ex*_ on the contact. This means that *d*_*o*_ now follows via *f*_*ex*_ = *f*_*pp*_(*d*) + *f*_*matrix*_(*d*). The authors also assume that *f*_*ex*_ is independent of *d* (we return to this important assumption below). In Fig. 1(d) *f*_*ex*_ is indicated by the horizontal line. In this sketch, where *f*_*ex*_ is close to zero, the large solid circle indicates the position of *d*_*o*_. When *f*_*ex*_ becomes increasingly negative (down arrow), the circle shifts continuously along the solid curve towards point A. When the circle passes A, however, it discontinuously jumps to the far right branch of the solid line. Subsequently the solid circle slides down along this line, i.e. *d*_*o*_ again increases continuously. When *f*_*ex*_ is reversed, the solid circle slides back up along the same branch until it passes point B. Here a second discontinuous jump returns it to the branch on which it started. Figure 1(d) shows the resulting path in the *f*_*ex*_-*d*_*o*_-plane for a full cycle. This means that the contact is both opened and closed. The shaded area enclosed by the path is *W*_*loss*,*j*_, which is the work dissipated into the polymer matrix during the two ‘jumps’. Note that this mechanism is non-destructive, i.e. it is mechanically (but not thermodynamically) reversible. Notice also that a similar mechanical instability occurs in surface probe microscopy, where it is known as ‘jump-to-contact’ (e.g. ref. 17). In cell biology an analogous mechanism is studied in the context of cell adhesion (e.g. ref. 18).

Thus far we have not mentioned the viscous loss, *W*_*loss*,*v*_, which contributes to the total energy dissipation in addition to the above *W*_*loss*,*j*_, i.e. the total loss is *W*_*loss*_ = *W*_*loss*,*v*_ + *W*_*loss*,*j*_. The viscous loss, concentrating on the viscous loss from the polymer in the vicinity of the particle surfaces, does depend on frequency. Here this is the rate of change of *f*_*ex*_. In the context of rolling resistance the attendant (macroscopic) frequency range is between 10 to 100 Hz. The ‘jump’ itself, however, is governed by the material’s elasticity and involves much larger frequencies. A full analysis of the contact model must include this. In particular, we must disentangle the relative importance of *W*_*loss*,*j*_ compared to *W*_*loss*,*v*_.

### Viscous loss vs. jump-loss

**Figure 2:**
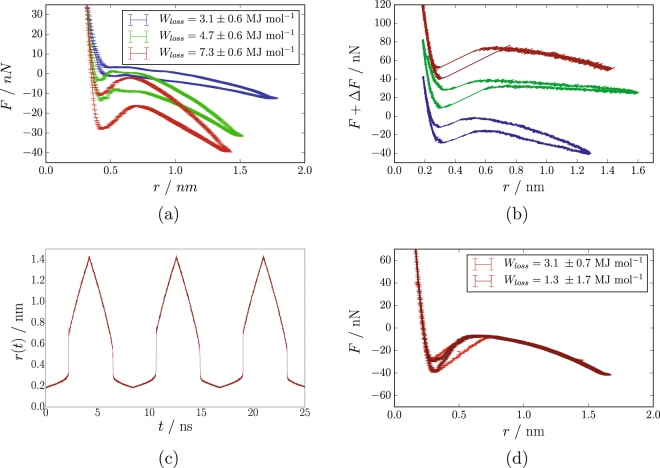
(**a**) Inter particle force-vs-inter particle distance for three different particle diameters, *D*, (blue: 2.1 nm, green: 4.2 nm, and red: 6.3 nm) at 300 K. (**b**) Single-cycle force curves for the largest particles at different temperatures, *T*, (blue: 300 K, green: 500 K, and red: 800 K). For better comparability the higher temperature curves are shifted vertically by Δ*F* = 40 nN (*T* = 500 K) and Δ*F* = 80 nN (*T* = 800 K), respectively. (**c**) Position, *r*, of the right particle during three subsequent cycles vs. time, *t*, at *T* = 800 K. (**d**) Force curves obtained with springs of different stiffness. Light red: *k* = *k*_0_; dark red: *k* = 4*k*_0_.

Figure 2(a) shows force-vs-distance curves for three different particle sizes obtained at 300 K. Notice that we measure the force between two silane-covered silica particles embedded in a sulphur-crosslinked polyisoprene matrix using molecular dynamics computer simulations as explained in the methods section. Each curve is an average over five subsequent cycles, excluding an initial cycle because of its transient character. The repulsion caused by the immediate contact of the particle surfaces occurs roughly at the same value of *r*, which here is a convenient radial coordinate. The lower section of each of the paths is obtained during the opening of the contact, whereas the upper section is obtained during closing of the contact.

In the case of the smallest particles the overall force curve, aside from its repulsive part, is rather flat. Because of the small particle size the elastic restoring force is barely noticeable. There are simply not enough polymer chains being deformed. The area enclosed by the force-vs-distance curve is *W*_*loss*_. In the case of the smallest particles, *W*_*loss*_ is entirely due to the viscous dissipation during particle movement. The attendant result in the case of the largest particles is different. We obtain a rather pronounced *S*-shape. This is because the repulsion at small *r* is followed by a trough, signalling attraction. The ‘naked’ contact area (cf. the section on methodology below) causes an additional ‘stickiness’ between the particles, which increases with particle size due to direct silica-silica interaction. In conjunction with the restoring force of the polymer matrix, which is now contributed by more chains in comparison to the small particles, the attendant interaction potential gives rise to an additional hysteresis during cyclic deformation of the contact. Thus, the overall shape, aside from the hysteresis, corresponds to our expectation as discussed in the context of Fig. 1(d). But where is the jump?

The next panel, Fig. 2(b), compares three force-vs-distance curves obtained with the largest particles at three different temperatures. Here, in contrast to the previous figure, each curve is the result of a single cycle, i.e. it is not an average over multiple cycles. Note that all curves do exhibit linear section, at intermediate *r*-values, devoid of the usual scatter. Figure 2(c) shows the attendant *r*-position of the right particle versus time, *t*, for *T* = 800 K. This figure contains the data obtained during three consecutive cycles. The jump-like particle displacements, signalling the opening and the closing of the contact during each cycle, are clearly discernible. However, if we compare for instance the uppermost curve in Fig. 2(b) to the sketch of the theoretically expected jump-hysteresis in Fig. 1(e), we do notice a difference. The area of the hysteresis obtained in the simulation appears rotated. This rotation or tilt occurs because, contrary to the original simplifying assumption in ref. 15, there is an appreciable dependence of the external force on *d* (or *r*). Notice that in the simulation the external force is different at point A, referring to Fig. 1(e), in comparison to its value at point A’. The same is true if points B and B’ are compared. Thus, the external force acting on the contacts in a realistic system, which here is exerted by the force gauge (cf. the methods section), is reduced during the jump.

In order to separate the viscous loss from the jump-loss we introduce springs of different stiffness in the force gauge. Figure 2(d) compares two force curves obtained using significantly different spring constants. The temperature is 800 K and each of the two curves is an average over five consecutive cycles. One curve, obtained with a soft spring (spring constant *k*_0_), is identical to the 800 K-result already shown in Fig. 2(b). The other curve is obtained under identical conditions but with a much stiffer spring. Note that a sufficiently stiff spring does prevent the jump, because it ensures full control over the particle position independent of the inter-particle force (somewhat analogous to a ‘towing bar’ in comparison to a ‘towing rope’). If the jump does not occur, then there is no contribution due to *W*_*loss*,*j*_. Consequently the total loss becomes *W*_*loss*_ = *W*_*loss*,*v*_.

**Figure 3:**
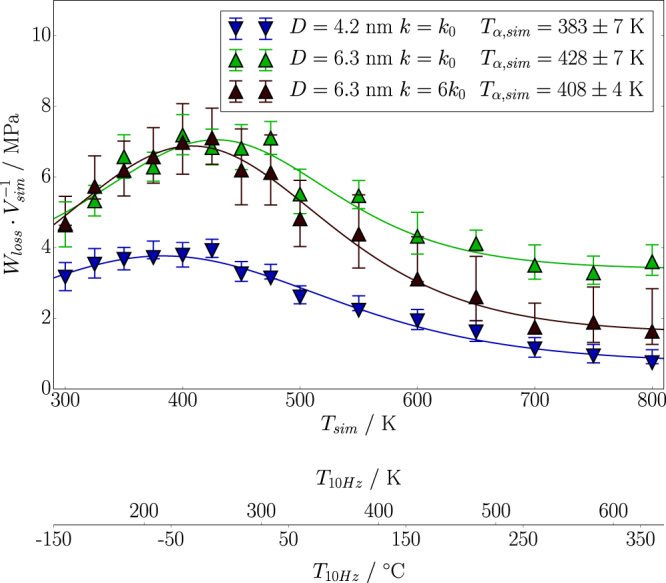
*W*_*loss*_ vs. *T* for the particle sizes and spring constants indicated in the figure. The lines are meant to guide the eye. Notice that *T*_*α*,*sim*_ is the peak position as obtained in the simulation.

In Fig. 3 we investigate the separation of the two types of energy dissipation systematically. The figure shows the total loss, *W*_*loss*_, (normalised to the simulation volume) vs. temperature. The lowest set of data points is obtained using particles with diameter *D* = 4.2 nm, whereas the other two data sets are obtained with the largest particles, i.e. *D* = 6.3 nm. All three resulting curves posses the same shape, i.e. a maximum occurring around 400 K is followed by a monotonous decrease at higher temperatures.

Notice that there is a qualitative similarity between the behaviour of tan *δ* as depicted in Fig. 1(a) and *W*_*loss*_/*V*_*sim*_ shown here. This is not by accident. The loss modulus, *μ*′′, of a linear elastic material is related to the dissipated energy density in the same material, *w*, via *w* = *πμ*′′*u* ^2^, where *u* is the strain amplitude (cf. ref. 8). Even though filled systems are non-linear, as pointed out above, this relation may and routinely is applied to the analysis of rheometric data, which means that it becomes a definition of the loss modulus rather than being the result of a derivation. In the sense of this ‘definition’ our *W*_*loss*_/*V*_*sim*_ is closely related to the loss modulus. It is important to note that *μ*′′ by itself may be used as an indicator for rolling resistance^14^, albeit one of somewhat inferior quality compared with tan *δ* = *μ*′′/*μ*′. Thus, for the present purpose, which is a ‘proof of principle’ rather than a quantitative calculation, it is entirely permissible to connect *W*_*loss*_/*V*_*sim*_ to the rolling resistance.

**Figure 4:**
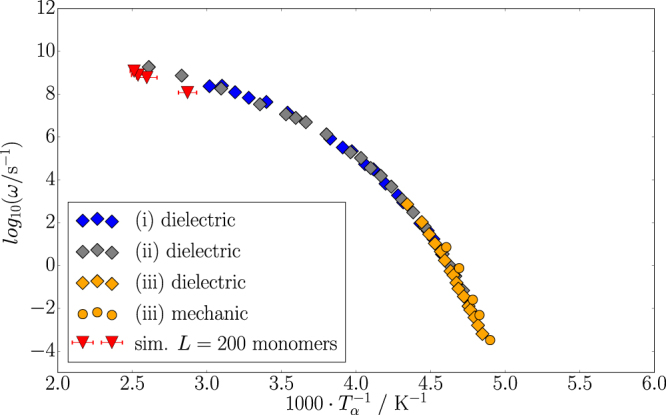
Relation between frequency and temperature of the segment relaxation peak (*α*-process) for polyisoprene. Experimental molecular weights are between 97.0 kg mol^−1^ to 504.0 kg mol^−1^. This figure is adopted from ref. 19.

A serious obstruction to the direct comparison between simulation and technical data, however, is the huge difference between time scales separating molecular simulations from the 10 to 100 Hz frequency range relevant to rolling resistance (cf. above). The particle velocity in the simulations, excluding the jumps, is about 0.5 m/s, i.e. the attendant cycle frequencies are in the GHz-range. In ref. 19 we have tracked the position of the segment relaxation peak, which is the aforementioned peak in Fig. 3, along the temperature axis within the narrow frequency range accessible to our simulations. Due to the small particle size in these simulations the calculated loss is almost entirely generated within the polymer. The attendant simulation results and complementary experimental results, obtained via dielectric relaxation spectroscopy as well as mechanical methods, are shown in Fig. 4 (which we reproduce here from ref. 19). The significance of this figure is twofold. First it confirms that our simulations reproduce the correct segment dynamics. And secondly the figure permits to relate the simulation results via the temperature-time superposition principle to technically more relevant frequencies. If we are interested in carrying our simulation results for *W*_*loss*_/*V*_*sim*_ over to a frequency of 10 Hz, we can proceed as follows. Using the master curve in Fig. 4, we determine the position of the segment relaxation peak along the temperature axis for this particular frequency, *T*_*α*,10Hz_. The temperature position of the same peak in the simulation is *T*_*α*,*sim*_. In this case the difference between the two temperatures is roughly 170 K. We may now convert every temperature value along the (simulation) temperature axis in Fig. 3 to a corresponding temperature when the frequency is 10 Hz simply by subtracting the difference *T*_*α*,10Hz_ − *T*_*α*,*sim*_. The result is the second temperature axis in Fig. 3, where the experimental temperature is given in units of Kelvin as well as degree Celsius.

Finally, we compare the two upper data sets in Fig. 3, which are obtained for the largest particles in both cases, using a soft and a hard spring, respectively. The former allows the jump to occur, i.e. *W*_*loss*_ = *W*_*loss*,*v*_ + *W*_*loss*,*j*_ whereas the second essentially prevents it, i.e. *W*_*loss*_ ≈ *W*_*loss*,*v*_. At temperatures near and below the maximum of the segment relaxation peak we do not observe much of a difference. This is because the dominant contribution to *W*_*loss*_ is viscous loss. Above the maximum of the segment relaxation peak, however, the results differ. As the temperature increases the viscous loss diminishes, whereas the loss caused by the spontaneous opening and closing of the contact persists and finally dominates.

We want to finish with a remark. Here we study a model of a single filler-filler contact along a load bearing path. Quantitative computations must include the filler network as a whole. This also encompasses the relation between filler distribution or network morphology, the system’s chemical composition and material preparation steps. Currently such a program does not appear to by a feasible approach, at least on the molecular level. Nevertheless, the present work describes a key dissipative mechanism occurring in filled elastomers. To the best of our knowledge, it is the only one described thus far at this level of detail.

## Methodology

### Modelling

**Figure 5:**
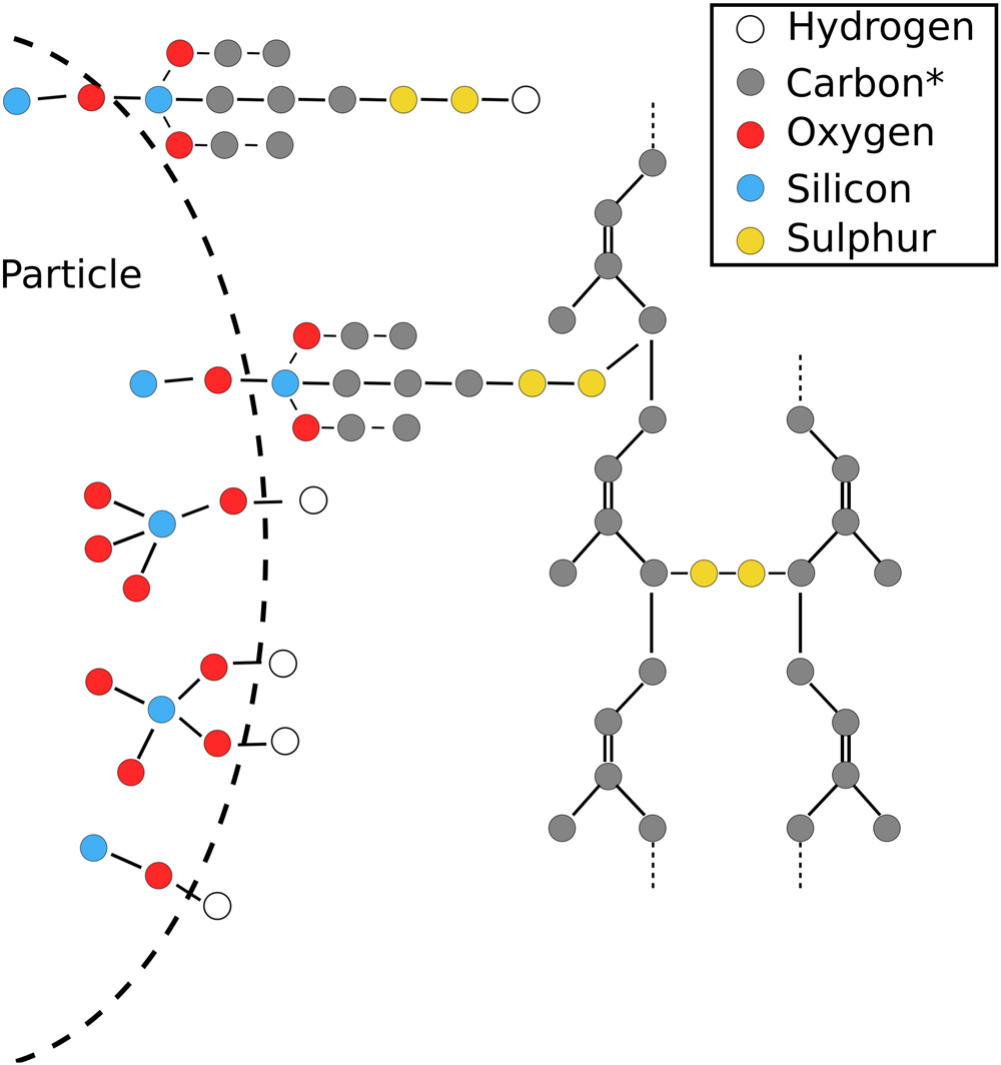
Overview depicting the variable chemical composition in the system. (*united atom).

The simulation setup follows in part the approach already discussed in refs 19, 20. A partial simulation snapshot is included in Fig. 1(b), showing two silica particles in close proximity embedded inside a cis-1,4-polyisoprene matrix. The silica particles are cut from *β*-cristobalite. Subsequently the surface is saturated with hydroxyl groups, which are then partially replaced by [3-(triethoxysilyl)propyl] disulfide (TESPT) during silanization (compare Fig. 5). In the present work the average silane density is *σ* = 2.7 nm^−2^. Notice that the immediate contact area between the two silica particles is kept free of silanes. The polymer molecular weight in the present work is always *M*_*w*_ = 13.6 kg mol^−1^(monodisperse). Polymers are covalently cross-linked at random via disulfide bonds. The same bonds also link the silane to the polymer. A schematic of the chemical setup is depicted in Fig. 5. Overall this setup includes all basic elements of the real material. The final systems do consist of about 10^5^ atoms. Their overall sulphur content is 15 phr, i.e. the mean distance between two crosslinks is smaller than the particle’s diameter. For the simulations we use the program package Gromacs 5.0.5^21^. Details regarding the force field, its parameterization and validation are presented in the aforementioned references.

The force between the two particles, *F*_*contact*_, is measured using a harmonic spring connected to the right particle, whereas the left particle’s position is fixed. Notice that *F*_*contact*_ includes the direct particle-particle interaction as well as the particle-particle interaction due to free energy change in the surrounding polymer matrix. The other end of the spring is connected to a virtual particle, which does not interact with any other atom in the system. For every position of the virtual particle there will be a corresponding position of the right silica particle satisfying the force equilibrium *F*_*spring*_ = *F*_*contact*_. By moving the virtual particle periodically back and forth on a line connecting the particles’ centres we are able to obtain the force-vs-displacement curves presented in this article.

### Statistical errors

During each cycle *i* we obtain sets of data, ${\{{F}_{spring,i}^{(\alpha )}(t);{r}_{i}^{(\alpha )}(t)\}}_{i}^{\alpha }$, where we distinguish between the directions of motion of the virtual particle, *α* = +/−. Notice that *r* is the radial coordinate of the second particle’s centre relative to the first particle’s centre minus the diameter of the utilised particles. We ignore the first cycle because of its transient character. In the next step we calculate the average force curves over all opening and all closing paths separately.

1 $${\overline{F}}^{(\alpha )}(r^{\prime} )=\frac{\sum _{i=1}^{n}\sum _{t}{F}_{i}^{(\alpha )}(t)\cdot \delta ({r}_{i}^{(\alpha )}(t)-r^{\prime} )}{\sum _{i\mathrm{=1}}^{n}\sum _{t}\delta ({r}_{i}^{(\alpha )}(t)-r^{\prime} )}.$$

The results are sorted into a histogram, i.e. *r*′ → [*r*′ − *dr*, *r*′ + *dr*] with *dr* = 0.005 nm. The quantity *σ* ^(*α*)^(*r*′) is the standard deviation of ${\overline{F}}^{(\alpha )}(r^{\prime} )$ divided by $\sqrt{n-1}$, which is used to specify the error bars. We obtain the dissipated energy via

2 $${W}_{loss}={\int }_{-}{\overline{F}}^{(-)}(r)dr+{\int }_{+}{\overline{F}}^{(+)}(r)dr.$$

For the dissipation we also calculate upper and lower bounds using

$${W}_{loss}^{\pm }={\int }_{-}[{\overline{F}}^{(-)}(r)\pm {\sigma }^{(-)}(r)]dr+{\int }_{+}[{\overline{F}}^{(+)}(r)\mp {\sigma }^{(+)}(r)]dr$$

These values determine the error bars shown in Fig. 3, whereas for the errors in all other figures only $\pm {W}_{loss}^{+}$ is used.
