# Author Correction: A nano-mechanical instability as primary contribution to rolling resistance

**DOI:** 10.1038/s41598-019-48628-w

**Published:** 2019-08-28

**Authors:** Jan Meyer, Reinhard Hentschke, Jonathan Hager, Nils W. Hojdis, Hossein Ali Karimi-Varzaneh

**Affiliations:** 1 School of Mathematics and Natural Sciences, Bergische Universität, D-42097 Wuppertal, Germany; 2 Continental Reifen Deutschland GmbH, D-30419 Hannover, Germany

Correction to: *Scientific Reports* 10.1038/s41598-017-11728-6, published online 12 September 2017

This Article contains errors in Reference 17, which was incorrectly given as:

Rodrigues, M. S., Costa, L., Chevrier, J. & Comin, F. Why do atomic force microscopy force curves still exhibit jump to contact? *Applied Physics Letters* **101**, 203105 (2016).

The correct reference is listed below as reference 1:
